# Nurses’ perspectives on human rights when coercion is used in psychiatry: a systematic review protocol of qualitative evidence

**DOI:** 10.1186/s13643-019-1224-0

**Published:** 2019-12-09

**Authors:** Pierre Pariseau-Legault, Sandrine Vallée-Ouimet, Marie-Hélène Goulet, Jean-Daniel Jacob

**Affiliations:** 1 Department of Nursing Sciences, Université du Québec en Outaouais, 5 rue Saint-Joseph, Saint-Jerome, Québec, J7Z 0B7 Canada; 2 Department of Nursing Sciences, Université du Québec en Outaouais, 5 rue Saint-Joseph, Saint-Jerome, Québec, J7Z 0B7 Canada; 3 Faculty of Nursing, Université de Montréal, Montréal, Canada; 4 School of Nursing, Faculty of Health Sciences, University of Ottawa, Ottawa, Canada

**Keywords:** Nursing, Human rights, Psychiatry, Mental health, Coercion, Meta-ethnography, Qualitative research

## Abstract

**Background:**

The World Health Organization describes the perpetuation of human rights violations against people with mental health problems as a global emergency. Despite this observation, recent studies suggest that coercive measures, such as seclusion, restraints, involuntary hospitalization, or involuntary treatment, are steadily or increasingly being used without proof of their effectiveness. In nursing, several literature reviews have focused on understanding nurses’ perspectives on the use of seclusion and restraints. Although many studies describe the ethical dilemmas faced by nurses in this context, to this date, their perspectives on patient’s rights when a broad variety of coercive measures are used are not well understood. The aim of this review is to produce a qualitative synthesis of how human rights are actually integrated into psychiatric and mental health nursing practice in the context of coercive work.

**Methods:**

Noblit and Hare’s meta-ethnographic approach will be used to conduct this systematic review. The search will be conducted in CINAHL, Medline, PsycINFO, ERIC, and Scopus databases, using the PICo model (Population, phenomenon of Interest, Context) and a combination of keywords and descriptors. It will be complemented by a manual search of non-indexed articles, gray literature, and other applicable data sources, such as human rights related documents. Qualitative and mixed-method study designs will be included in this review. Empirical and peer-reviewed articles published between 2008 and 2019 will be selected. Articles will be evaluated independently by two reviewers to determine their inclusion against eligibility criteria. The quality of the selected papers will then be independently evaluated by two reviewers, using the Joanna Briggs Institute’s Checklist for Qualitative Research. Data extraction and content analysis will focus on first- and second-order constructs, that is, the extraction of research participants’ narratives and their interpretation.

**Discussion:**

This review will provide a synthesis of how psychiatric and mental health nurses integrate human rights principles into their practice, as well as it will identify research gaps in this area. The results of this review will then provide qualitative evidence to better understand how nurses can contribute to the recognition, protection, and advocate for human rights in a psychiatric context.

**Systematic review registration:**

PROSPERO, CRD42019116862

## Background

The perpetuation of human rights violations in psychiatric settings is described as a global emergency by the World Health Organization [1]. Although regional differences exist, this “global crisis” affects all jurisdictions [2, 3]. Recent studies demonstrate that coercive measures, such as seclusion, physical or chemical restraints, involuntary treatment, and involuntary hospitalization, are steadily or increasingly being used in many jurisdictions [4] without clear evidence of their effectiveness when compared with treatments offered on a voluntary basis [5, 6].

The Merriam Webster dictionary defines coercion as the act of “compel(ling) to an act or choice” or “achiev(ing) by force or threat” [7]. Coercion can be either formal, informal, and/or perceived. Formal coercion refers to regulated, justified, and documented forms of coercion. Examples of formal coercion include involuntary hospitalization and/or treatment. Informal coercion refers to other forms of actions that use strategies to act against the patient’s will that do not involve the use of legal provisions. Examples of informal coercion include threats and deception [8]. Perceived coercion refers to the subjective experience or appreciation (generally by the patient himself) of what is/what is not coercion [9]. Examples of perceived coercion include feelings of injustice and unfairness [8].

Recent research shows the harmful effects of coercive measures on people with mental health problems. Although coercive measures are sometimes described as providing a sense of control and safety for patients, many of them also describe feelings associated with fear, trauma, and disempowerment when experiencing these measures [10–12]. In effect, the literature consulted prior to this review would suggest that involuntary hospitalization is associated with a higher risk of suicide during hospitalization [13] and that perceived coercion in a psychiatric setting also increases the risk of post-discharge suicide attempts [14].

It is also suggested that it is rather common factors, such as the intensity of services [6, 15] and the quality of the therapeutic relationship between the person and health professionals [10, 11, 16, 17], that would influence the recovery of the person under coercive measures. In such a context, managerial approaches to risk management (which often justify the imposition of coercive measures) should be complemented and calibrated by an emphasis on relational approaches to care [10].

Nurses, like many health professionals, have an important responsibility to protect, recognize, and defend the rights of people with mental health issues, including human rights [18, 19]. However, the evidence suggests that many health professionals sometimes exceed the limits imposed by law [20], may underestimate informal coercion [21], and should be educated about its use and potential adverse effects given its estimated prevalence in psychiatric settings between 29 and 59% [22]. It is also suggested that health professionals can use formal or informal coercion as a means of leverage and control rather than as a last resort intervention [22, 23]. Knowledge of the laws governing the use of these exceptional measures in psychiatry, that have a direct impact on the recognition of the rights of persons under coercion, is considered inconsistent between different types of health professionals and generally highly insufficient [24].

Little is known about how human rights are understood, integrated into practice, and participate in the experience of the health professionals responsible for implementing these measures. In nursing, several literature reviews focus on decision-making [25], ethical challenges [9], or the quality of care provided under coercion [26]. However, to date, no systematic literature review has qualitatively synthesized the clinical relevance of human rights in psychiatric and mental health nursing practice. While various systematic and non-systematic literature reviews focus on the practice of nurses [9, 27, 28], most of them are specific to a jurisdiction [28] or intervention [27]. A recent systematic review aimed to identify the ethical challenges that arise when coercion is used in mental health care but considered human rights as an exclusion criterion [9]. To our knowledge, no literature review directly addresses human rights issues in the practice of psychiatric and mental health nurses.

## Aims and objectives

This systematic literature review will focus on nurses’ perspectives on human rights when coercion is used in various psychiatric contexts. The aim of this review is to produce a qualitative synthesis of how human rights are actually integrated into nursing practice in the context of coercive work. The first objective of this review is to describe psychiatric and mental health nurses’ contemporary understanding of human rights in psychiatry. The second objective of this review is to produce an explanatory framework of how human rights are recognized, protected, or challenged in psychiatric and mental health nursing. The main research question is as follows: What are the perspectives of nurses working in adult psychiatry regarding the patient’s rights when coercion is used? What we mean by “perspectives” will be defined in the “Search strategy” section.

## Methodology and methods

This systematic literature review protocol is registered with the International prospective register of systematic reviews (PROSPERO, CRD42019116862). The Preferred Reporting Items for Systematic review and Meta-Analysis Protocols (PRISMA-P) is included as Additional file 1 to this protocol to report how the review was conducted [29]. After the data extraction, the meta-ethnographic approach of Noblit and Hare [30] will be used to synthesize the data. The reviewers will then refer to the eMERGe reporting guidance [31] to report on the analytic synthesis process followed to produce the meta-ethnography and qualitative findings. This research protocol integrates the phases 1 (selecting meta-ethnography and getting started), 2 (deciding what is relevant), and 3 (reading included studies) of the meta-ethnography [31] since they are specifically related to the systematic literature review. During the literature review process, all identified articles will be managed with the EndNote® software.

### Study design

Meta-ethnography is a “theory-based and potentially theory-generating interpretive methodology for qualitative evidence synthesis” [31] developed in the field of sociology (p. 2). Noblit and Hare propose a seven-step approach for qualitative evidence synthesis: (1) selecting meta-ethnography and getting started, (2) deciding what is relevant, (3) reading included studies, (4) determining how studies are related, (5) translating studies into one another, (6) synthesizing translations, and (7) expressing the synthesis [30, 31]. This strategy allows for the development of interpretive explanations related to the extracted data and for a synthesis of these interpretive explanations in an explanatory framework. Noblit and Hare’s methodological approach is frequently used in health sciences to provide a qualitative synthesis of patients’ or health professionals’ experiences of a given phenomenon [32, 33]. Since the objectives of this systematic review is to synthesize and provide an explanatory framework of how human rights are integrated in the practice of psychiatric and mental health nurses in the context of coercive work, the meta-ethnographic approach appeared as the most appropriate methodology for this study. Although this approach is generally appropriate for a small number of qualitative studies, the authors of this protocol and literature review will draw on Toye et al.’s [34] guidance if needed to ensure that the synthesis of a larger number of studies “remain(s) firmly grounded in the [selected] primary qualitative studies” (p. 3).

### Search strategy

The research strategy was developed in an inductive manner during the preliminary research phase. The greatest challenge in this step was to formulate a strategy to identify contemporary empirical and qualitative articles discussing psychiatric and mental health nurses’ perspective on human rights when coercive measures are used. During this stage, different search strategies were tested in order to decide what was relevant for the meta-ethnography [30, 31]. It was determined that the phenomenon of interest in this review was coercion, from which various human rights issues may arise.

The PICo method for qualitative research (Population [nurses], phenomenon of Interest [coercion], Context [psychiatry and mental health]) was then used to formulate the research strategy [35]. Various articles addressing the phenomenon of coercion in a psychiatric context were first identified by manual research. This search facilitated the identification of key concepts in the final research strategy. For example, it was possible to apply the concept of coercion in different ways by identifying keywords and descriptors of situations typical to the psychiatric context (e.g., seclusion, restraints, involuntary hospitalization, and treatment). Similarly, terms such as involuntary hospitalization varied among authors and jurisdictions. During this stage, it was also possible to clarify what was meant by nurses’ “perspectives” (see Table 1). A university librarian was also consulted to support the development of the search strategy. The keywords and descriptors included in the research strategy are presented in Table 1.

**Table 1:** Key words and descriptors of the search strategy

Population—nurses’ perspectives	AND	Phenomenon of interest—coercion	AND	Context— psychiatry
Nurs* Knowledge Opinion* Attitude* View* Belie* Behavior* Practice* Intervention* Reaction* Perspective* Perception* Experience* Decision* Culture*		(MH "Restraint, Chemical") (MH "Restraint, Physical") (MH "Coercion") (MH "Involuntary Commitment") Coerc* Restraint* Isolation* Seclusion* OR Involuntary Compulsory Coerc* Forced Mandat* Order* Assisted Supervised Extended Conditional AND Hospitali#ation* Admission* Commitment* OR Treatment* Care* Medication* Leave* Discharge*		(MH "Psychiatry") (MH "Mental Health") (MH "Psychiatric Nursing") Psychia* Mental health Forensic

*Major descriptors will be added to these keywords and will vary according to the databases consulted. This table represents the search strategy for CINAHL Database

### Information sources

The search will be conducted in CINAHL, Medline, PsycINFO, ERIC, and Scopus databases. Since the aim of this meta-ethnography is to provide a synthesis of the contemporary perspective of psychiatric and mental health nurses on human rights when coercion is used in psychiatry, only articles published between 2008 and 2019 will be considered. The literature review will also be updated prior to the publication of the results. This review will be complemented by a manual search of non-indexed articles, gray literature, and other applicable data sources, such as human rights–related documents. Qualitative and mixed-methods study designs will be included in this review. Quantitative study designs will be excluded from this review.

## Search process

The systematic literature review will be carried out independently by two reviewers (PPL and SVO). The selection of articles will be carried out in three stages, namely by reading their title and abstract, by reading the articles according to the pre-established eligibility criteria, and by evaluating their quality. The following section describes the measures used by the reviewers to systematize this approach and avoid the risk of bias.

### Selection of primary studies

This literature review will include peer-reviewed, empirical and qualitatively designed articles written in English or in French, as well as non-indexed articles, gray literature, and other applicable data sources found by manual search, such as human rights–related documents. In order to be included in this review, studies must also clearly address the psychiatric and mental health nurses’ perspective on coercion. The criteria for the inclusion of articles are as follows: nurses, working in psychiatry/mental health, using coercion, qualitative design. The exclusion criteria for articles are as follows: child and adolescent psychiatry, geriatric psychiatry, learning disability nursing.

The titles and abstracts of the articles identified by the research strategy will be evaluated independently by two reviewers using the PICo method for qualitative research. The selected articles will then be read in order to be selected or excluded against eligibility criteria. The quality of the selected papers will finally be independently evaluated by two reviewers, using the Joanna Briggs Institute’s Critical Appraisal tool—Checklist for Qualitative Research [36]. Prior to this step, reviewers will become familiar with this tool by independently assessing the quality of a minimum of 5 articles similar to those identified in the early stages of the literature review. This tool will allow an analysis of the selected literature based on scientific criteria specific to qualitative research. This evaluation will be useful in identifying the most significant articles in relation to the research question [34].

This quality analysis will be completed by a general assessment of confidence in the concepts that emerged from the meta-ethnography using CERQual’s assessment of confidence for individual review findings from qualitative evidence syntheses [37]. Along with the quality assessment of each selected papers using the Joanna Briggs Institute’s Critical Appraisal tool—Checklist for Qualitative Research [36], other components of the CERQUAL approach (relevance, coherence, and adequacy of data) will be assessed for each core concepts. A qualitative evidence profile including those components will be created [31, 37]. This summary table will make an overall assessment of confidence based on the reviewers’ assessment of the quality of each selected article, the applicability of the synthesized body of evidence to the phenomenon of interest (coercion), the study population (nurses) and the targeted context (psychiatry and mental health) (*relevance*), the *coherence* between the core concepts that have emerged from meta-ethnography and their underlying studies, and a general appreciation of the importance of these concepts in relation to the data (first-order and second-order constructs) collected (*adequacy of the data*) [37].

During this process, any difference of opinion between the two reviewers will be resolved by consensual agreement. In a case where there is no consensus, a third reviewer will be involved to make a final decision (MHG or JDJ). The trustworthiness of this study will be established by the methodological transparency adopted by the reviewers; by using standardized tools such as the PRISMA-P checklist (see Additional file 1) [29], the Joanna Briggs Institute’s Critical Appraisal tool—Checklist for Qualitative Research [36], and the CERQual’s assessment of confidence for individual review findings from qualitative evidence syntheses [37]; and by the differentiation of first- and second-order constructs from third-order constructs during the final stage of the analysis. First-order constructs refer to the participants’ narratives in the selected studies, while second-order constructs refer to the researchers’ interpretation of them [31, 34]. Third-order constructs refer to the synthesis of qualitative data performed by the reviewers. To avoid any conflict of interest during the review process, articles previously written by one reviewer will be independently and anonymously evaluated by another reviewer.

### Data extraction

General descriptive data will be extracted from the selected literature (author(s), year of publication, country of origin, language, title, aim, study design and methodology, number, and characteristics of participants). If possible, data related to the settings in which the coercion takes place (for example, hospital care, community care), as well as the type of coercion used (for example, use of restraint, seclusion, or involuntary treatment) will also be extracted. Since the analysis process is iterative, inductive, and based on qualitative data, it is possible that new clusters of data emerge during data extraction and synthesis. Following the approach suggested by Noblit and Hare [30, 31], qualitative data from the selected studies’ results will also be extracted. More specifically, first- and second-order constructs will be extracted and analyzed to produce an interpretive synthesis of the data. Thus, the extraction of this type of data will focus on identifying the themes, metaphors, or central concepts of each study [31, 34].

### Data synthesis

The meta-ethnographic approach used will require a thorough reading and re-reading of the selected studies in order to identify key concepts. All of these studies will be inserted into NVivo software which will facilitate the extraction of general descriptive and qualitative data, as well as the process of immersion within the data [34]. Thus, the coding of data and the identification of key concepts of the selected studies will be carried out through this software. If necessary, these data will also be aggregated in the form of an excel table to facilitate the synthesis and description of the results of the literature review. Data synthesis will be collaborative and carried out by the two main reviewers (PPL and SVO). The data synthesis will also be reviewed by MHG and JDJ until consensus is reached among the four reviewers.

The synthesis of the qualitative data extracted in the previous step will consist in identifying the key concepts of the selected studies and determining how the different studies are associated with each other, to the research question and the phenomenon of interest [31, 34]. These concepts must above all remain faithful to the description of human rights during coercive work as it is experienced by psychiatric and mental health nurses [34]. This synthesis, which consists of “translating studies into one another” (p. 9), will integrate reciprocal (converging) and refutational (diverging) data [31].

The collaborative process in which the reviewers will engage will allow a consensus to be reached on the interpretation of first- and second-order constructs [34]. The general descriptive data and qualitative data from each study will be compared with each other so that the approach remains sensitive to the context of the data collected. Subsequently, these concepts will be synthesized to produce an explanatory framework of how human rights are integrated into the practice of psychiatric and mental health nurses.

## Discussion

Current research hypothesizes that it is primarily the intensity of services [6, 15] and the quality of the therapeutic relationship [10, 17], rather than coercion, that have a positive effect on the person’s recovery. Anecdotal evidence suggests the positive impact of human rights-based approaches to the recovery of people with mental health issues [3, 38, 39]. In order to further explore the potential impact of these approaches, it seems necessary to map the current state of practices of health professionals who share responsibility for implementing coercive measures. This qualitative research review will provide a better understanding of how human rights issues influence or are considered in the daily practice of nurses when using coercion in psychiatry.

Over the past decade, several systematic reviews have documented the perspective of psychiatric and mental health nurses in terms of the decision-making process they engage [25], the ethical dilemmas they face [9], and the quality of care they provide in a coercive context [26]. However, few studies focusing on the provision of nursing care in a coercive context have adopted a human rights perspective [40]. This is surprising, since coercive measures are considered by many as a significant violation of human rights and should therefore be used as a last resort [41]. A recent study by the first and third author [42] suggests many difficulties in applying coercive measures like involuntary treatment in the psychiatric setting, such as the involvement of family caregivers and the maintenance of the therapeutic alliance between service users and health professionals. Involuntary treatment is also sometimes perceived as promoting better access to mental health while inducing a feeling of fear for the person concerned [43]. A recent meta-ethnography also demonstrates that the main themes associated with the experience of people affected by seclusion in psychiatry are the feeling of being “admitted to prison” and deprived of their rights [44]. These findings are troubling for the recognition, protection, and promotion of human rights in the psychiatric context and require further exploration.

To our knowledge, this will be the first literature review combining a meta-ethnographic approach and a human rights perspective to examine the attitudes, experience, and practices of psychiatric and mental health nurses related to coercive work. By including a human right perspective, this systematic review will identify research areas that need further exploration and contribute to the development of a research agenda related to coercion and human rights in psychiatric and mental health work. The quality assessment of the articles prior to their final inclusion will also provide an opportunity to discuss the challenges associated with this step when meta-ethnography is used as a methodological approach. It will also be possible to discuss the global methodological issues associated with qualitative research on this topic. The meta-ethnographic approach will be particularly useful in determining how human rights are conceptualized in the scientific literature reviewed. By distinguishing first-order and second-order constructs, it will promote a better understanding of the use and integration of these rights in nurses’ daily practice. The extraction and analysis of first-order and second-order constructs will provide a critical review of the scientific literature to identify how the work of nurses, who share the responsibility for implementing these coercive measures, is framed as a human rights issue. The results of this review will then provide qualitative evidence to better understand how nurses can contribute to the recognition, protection, and advocate for human rights in a psychiatric context.

## Limitations and strengths

Several limitations to this systematic literature review and meta-ethnography must be addressed. Since the data from this protocol target coercion, it is possible that the importance of different approaches to coercion prevention (e.g., de-escalation techniques) or postvention may be minimized. As this study focuses on the nurses’ perspectives, it is also possible that interdisciplinary or non-nursing specific studies may be excluded. This study is also limited by the contextual nature of human rights, the recognition of which differs from one jurisdiction to another. This study also presents different strengths. To our knowledge, although the principles of advocacy and social justice are central to nursing practice, no study has yet synthesized qualitative evidence on human rights issues in psychiatric and mental health nursing. In a context where it is suggested that relational approaches to care can at least partially address the issues raised by coercion [10, 17] and that rights-based approaches can facilitate the recovery process [3, 38, 39], this study is also an opportunity to better understand how these approaches can be integrated into current nursing practices.

## Supplementary information

**Additional file 1.** PRISMA-P 2015 Checklist.

## Data Availability

Not applicable.

## References

[1] World Health Organization. Mental health [Internet]. Available from https://www.who.int/mental_health/policy/legislation/en/

[2] Drew N, Funk M, Tang S, Lamichhane J, Chavez E, Katontoka S (2011). Human rights violations of people with mental and psychosocial disabilities: an unresolved global crisis. Lancet..

[3] Porsdam Mann S, Bradley VJ, Sahakian BJ (2016). Human rights-based approaches to mental health: a review of programs. Health Hum Rights..

[4] Sashidharan S. P., Mezzina Roberto, Puras Dainius (2019). Reducing coercion in mental healthcare. Epidemiology and Psychiatric Sciences.

[5] Kisely SR, Campbell LA, O’Reilly R (2017). Compulsory community and involuntary outpatient treatment for people with severe mental disorders. Cochrane Database Syst Rev..

[6] Rugkasa J, Burns T (2017). Community treatment orders: are they useful?. BJPsych Advances..

[7] Merriam Webster Dictionary. Coercing [Internet]. Available from https://www.merriam-webster.com/dictionary/coercing

[8] Szmukler G, Appelbaum PS (2008). Treatment pressures, leverage, coercion, and compulsion in mental health care. Journal of Mental Health..

[9] Hem MH, Gjerberg E, Husum T, Pedersen R (2018). Ethical challenges when using coercion in mental healthcare: a systematic literature review. Nurs Ethics..

[10] Verbeke E, Vanheule S, Cauwe J, Truijens F, Froyen B (2019). Coercion and power in psychiatry: a qualitative study with ex-patients. Soc Sci Med..

[11] Seed T, Fox JR, Berry K (2016). The experience of involuntary detention in acute psychiatric care : a review and synthesis of qualitative studies. Int J Nurs Stud..

[12] Wilson C, Rouse L, Rae S, Kar RM (2017). Is restraint a ‘necessary evil’ in mental health care? Mental health inpatients’ and staff members’ experience of physical restraint. Int J Ment Health Nurs..

[13] Large M, Smith G, Sharma S, Nielssen O, Singh SP (2011). Systematic review and meta-analysis of the clinical factors associated with the suicide of psychiatric in-patients. Acta Psychiatr Scand..

[14] Jordan J, McNiel D. Perceived coercion during admission into psychiatric hospitalization increases risk of suicide attempts after discharge. Suicide Life Threat Behav. 2019. 10.1111/sltb.12560. 10.1111/sltb.12560 31162700

[15] Barnett PB, Matthews H, Llyod-Evans B, Mackay E, Pilling S, Johnson S (2018). Compulsory community treatment to reduce readmission to hospital and increase engagement with community care in people with mental illness: a systematic review and meta-analysis. Lancet Psychiatry..

[16] Mullen R, Dawson J, Gibbs A (2006). Dilemmas for clinicians in use of community treatment orders. Int J Law Psychiatry..

[17] Corring D, O’Reilly R, Sommerdyk C (2017). A systematic review of the views and experiences of subjects of community treatment orders. Int J Law Psychiatry..

[18] Kelly BD (2015). Human rights in psychiatric practice: an overview for clinicians. BJPsych Advances..

[19] Gerber L (2018). Understanding the nurse’s role as a patient advocate. Nursing..

[20] Aasland OG, Husum TL, Førde R, Pedersen R (2018). Between authoritarian and dialogical approaches: attitudes and opinions on coercion among professionals in mental health and addiction care in Norway. Int J Law Psychiatry..

[21] Jaeger M, Ketteler D, Rabenschlagb F, Theodoridou A (2014). Informal coercion in acute inpatient setting—knowledge and attitudes held by mental health professionals. Psychiatry Res..

[22] Hotzy F, Jaeger M (2016). Clinical relevance of informal coercion in psychiatric treatment-a systematic review. Front Psychiatry..

[23] O’Reilly R, Corring D, Richard J, Plyley C, Pallaveshi L (2016). Do intensive services obviate the need for CTOs?. Int J Law Psychiatry..

[24] Holder SM, Warren C, Rogers K, Griffeth B, Peterson E, Blackhurst D (2018). Involuntary processes: knowledge base of health care professionals in a tertiary medical center in upstate South Carolina. Community Ment Health J..

[25] Laiho T, Kattainen E, Astedt-Kurki P, Putkonen H, Lindberg N, Kylmä J (2013). Clinical decision making involved in secluding and restraining an adult psychiatric patient: an integrative literature review. J Psychiatr Ment Health Nurs..

[26] van den Hooff S, Goossensen A (2014). How to increase quality of care during coercive admission? A review of literature. Scand J Caring Sci..

[27] Happell B, Harrow A (2010). Nurses’ attitudes to the use of seclusion: a review of the literature. Int J Ment Health Nurs..

[28] Ashmore R (2015). Section 5(4) (nurse’s holding power) of the mental health act 1983: a literature review. J Psychiatr Ment Health Nurs..

[29] Moher D, Shamseer L, Clarke M, Ghersi D, Liberati A, Petticrew M (2015). Preferred reporting items for systematic review and meta-analysis protocols (PRISMA-P) 2015 statement. Syst Rev..

[30] Noblit GW, Hare RD (1988). Meta-ethnography: synthesizing qualitative studies. In: Meta-Ethnography: Synthesizing Qualitative Studies.

[31] France EF, Cunningham M, Ring N, Uny I, Duncan EA, Jepson RG (2019). Improving reporting of meta-ethnography: the emerge reporting guidance. BMC Med Res Methodol..

[32] Bridges J, Nicholson C, Maben J, Pope C, Flatley M, Wilkinson C (2013). Capacity for care: meta-ethnography of acute care nurses’ experiences of the nurse-patient relationship. J Adv Nurs..

[33] Higginbottom GM, Hadziabdic E, Yohani S, Paton P (2014). Immigrant women’s experience of maternity services in Canada: a meta-ethnography. Midwifery..

[34] Toye F, Seers K, Allcock N, Briggs M, Carr E, Barker K (2014). Meta-ethnography 25 years on: challenges and insights for synthesizing a large number of qualitative studies. BMC Med Res Methodol..

[35] Stern C, Jordan Z, McArthur A (2014). Developing the review question and inclusion criteria. Am J Nurs..

[36] Joanna Briggs Institute. Checklist for Qualitative Research [Internet]. Available from https://joannabriggs.org/critical_appraisal_tools

[37] Lewin S, Glenton C, Munthe-Kaas H, Carlsen B, Colvin CJ, Gülmezoglu M, Noyes J, Booth A, Garside R, Rashidian A (2015). Using qualitative evidence in decision making for health and social interventions: an approach to assess confidence in findings from qualitative evidence syntheses (GRADE-CERQual). PLoS Med..

[38] Eaton J (2019). Human rights-based approaches to mental health legislation and global mental health. BJPsych Int..

[39] Puras D, Gooding P (2019). Mental health and human rights in the 21st century. World Psychiatry..

[40] Fawzy ME (2015). Quality of life and human rights conditions in a public psychiatric hospital in Cairo. International Journal of Human Rights in Healthcare..

[41] Steinert T (2017). Ethics of coercive treatment and misuse of psychiatry. Psychiatr Serv..

[42] Pariseau-Legault P, Goulet MH, Crocker, AG. Une analyse critique des effets de l’autorisation judiciaire de soins sur la dynamique relationnelle entre la personne visée et ses systèmes de soutien. Aporia. 2019;11(1):41–55.

[43] Goulet MH, Pariseau-Legault P, Côté C, Klein A, Crocker AG. Multiple stakeholders’ perspectives of involuntary treatment orders: a meta-synthesis of the qualitative evidence toward an exploratory model. International Journal of Forensic Mental Health. 2019. 10.1080/14999013.2019.1619000.

[44] Lindgren BM, Ringnér A, Molin J, Graneheim UH (2019). Patients’ experiences of isolation in psychiatric inpatient care: insights from a meta-ethnographic study. Int J Ment Health Nurs..

